# Identification of CREB3L1 as a Biomarker Predicting Doxorubicin Treatment Outcome

**DOI:** 10.1371/journal.pone.0129233

**Published:** 2015-06-25

**Authors:** Bray Denard, Andrea Pavia-Jimenez, Weina Chen, Noelle S. Williams, Harris Naina, Robert Collins, James Brugarolas, Jin Ye

**Affiliations:** 1 Department of Molecular Genetics, University of Texas Southwestern Medical Center, Dallas, Texas 75390, United States of America; 2 Department of Internal Medicine, Hematology-Oncology Division, University of Texas Southwestern Medical Center, Dallas, Texas 75390, United States of America; 3 Department of Developmental Biology, University of Texas Southwestern Medical Center, Dallas, Texas 75390, United States of America; 4 Department of Pathology, University of Texas Southwestern Medical Center, Dallas, Texas 75390, United States of America; 5 Department of Biochemistry, University of Texas Southwestern Medical Center, Dallas, Texas 75390, United States of America; 6 Kidney Cancer Program in Simmons Comprehensive Cancer Center, University of Texas Southwestern Medical Center, Dallas, Texas 75390, United States of America; Florida International University, UNITED STATES

## Abstract

**Background:**

Doxorubicin has been shown to inhibit proliferation of cancer cells through proteolytic activation of CREB3L1 (cAMP response element binding protein 3-like 1), a transcription factor synthesized as a membrane-bound precursor. Upon doxorubicin treatment, CREB3L1 is cleaved so that the N-terminal domain of the protein can reach the nucleus where it activates transcription of genes that inhibit cell proliferation. These results suggest that the level of CREB3L1 in cancer cells may determine their sensitivity to doxorubicin.

**Methods:**

Mice transplanted with 6 lines of renal cell carcinoma (RCC) were injected with doxorubicin to observe the effect of the chemotherapy on tumor growth. Immunohistochemistry and bioinformatics analyses were performed to compare CREB3L1 levels in types of cancer known to respond to doxorubicin versus those resistant to doxorubicin.

**Results:**

Higher levels of CREB3L1 protein are correlated with increased doxorubicin sensitivity of xenograft RCC tumors (p = 0.017 by Pearson analysis). From patient tumor biopsies we analyzed, CREB3L1 was expressed in 19% of RCC, which is generally resistant to doxorubicin, but in 70% of diffuse large B-cell lymphoma that is sensitive to doxorubicin. Doxorubicin is used as the standard treatment for cancers that express the highest levels of CREB3L1 such as osteosarcoma and malignant fibrous histiocytoma but is not generally used to treat those that express the lowest levels of CREB3L1 such as RCC.

**Conclusion:**

Identification of CREB3L1 as the biomarker for doxorubicin sensitivity may markedly improve the doxorubicin response rate by applying doxorubicin only to patients with cancers expressing CREB3L1.

## Introduction

Personalized medicine has gained much attention for cancer treatment and has set off a search for biomarkers that identify individuals who will benefit from a particular form of treatment. Doxorubicin has been extensively used to treat various cancers, but the response rate of the treatment for most cancers is low owing to the lack of such a biomarker to identify patients who will likely to benefit from the treatment. Doxorubicin has been assumed to exert its cytostatic effect through DNA damage, but a consistent correlation between doxorubicin-induced DNA breaks and secession of cell proliferation has not been established [1,2].

We have recently demonstrated that doxorubicin inhibits proliferation of cancer cells through proteolytic activation of CREB3L1 (cAMP response element binding protein 3-like 1). Unlike a typical transcription factor, CREB3L1 is synthesized as an inactive transmembrane precursor [3]. The protein contains a single transmembrane helix, with the N-terminal domain projecting into cytosol [4]. Upon doxorubicin treatment, CREB3L1 is proteolytically cleaved so that the N-terminal domain of the protein is translocated from membranes to the nucleus where it activates transcription of genes that inhibit progression of the cell cycle and those required for assembly of collagen-containing extracellular matrix [5]. Using tumor cells cultured *in vitro*, we reported that doxorubicin inhibited proliferation of cells expressing *CREB3L1* but not those in which the gene was not expressed, even though the drug was equally effective in triggering DNA damage in both cells [5]. These results suggest that the cytostatic effect of doxorubicin is caused by cleavage of CREB3L1 rather than DNA damage. In support of this conclusion, we reported previously that expression of a C-terminally truncated CREB3L1 resembling the cleaved nuclear form of CREB3L1 was sufficient to inhibit cell proliferation [4]. We thus surmised that CREB3L1 may be used as a biomarker to identify cancers that are likely to respond to doxorubicin treatment.

In the current study, we used a mouse xenograft model of renal cell carcinoma (RCC), which exhibits the same drug sensitivity as that displayed in patients [6], to demonstrate that doxorubicin inhibited growth of tumors that produced CREB3L1 but not those that did not express the protein. Using immunohistochemistry (IHC) and bioinformatics analyses, we showed that cancers known to be responsive to doxorubicin treatment such as diffuse large B-cell lymphoma (DLBCL) and osteosarcoma expressed higher levels of CREB3L1 than those known to be resistant to the treatment such as RCC. These results suggest that CREB3L1 may be used as a biomarker to identify cancers that respond to doxorubicin treatment.

## Results

In order to study the effect of doxorubicin on growth of xenograft tumors transplanted in mice, we first determined the dose of doxorubicin that can be safely applied to mice and compared it to that usually applied to human patients. Important pharmacokinetic parameters such as C_max_ and AUC of the mice receiving a single injection of doxorubicin at 5 mg/kg were lower than that of human patients receiving a single injection of doxorubicin at a typical clinical dose [7] (Table 1). However, doxorubicin appeared to be more toxic to mice than humans, as this dose of doxorubicin killed mice within a week after the injection. We then lowered the dose, and found that mice can tolerate up to 8 injections of doxorubicin at 0.75 mg/kg. Pharmacokinetic modeling analysis indicated that the cumulative therapeutic concentration of the drug under this condition was significantly lower than that in human patients treated with doxorubicin (Table 1, AUC).

**Table 1:** Comparison of doxorubicin pharmacokinetic parameters in mice and human patients.

		Mouse	Mouse	Human
Parameter	Unit	5 mg/kg IP	0.75 mg/kg IP	75 mg/m^2^ IV bolus
		Single injection	8 injections	Single injection
C_max_ (Concentration Max)	ng/ml	425	62.7	14,947
Α-Half-Life	hr	0.15	0.15	0.08
β-Half-Life	hr	15.3	15.3	2.4
ƴ-Half-Life	hr	n/a	n/a	33
AUC (Area Under the Curve)	hr×ng/ml	909	1072	2591

Mice were injected with a single dose of doxorubicin at 5 mg/kg intraperitoneally (IP), and plasma levels of doxorubicin were determined by LC-MS/MS as described in Materials and Methods. The data were fit to a 2-compartment model with bolus input (WinNonlin Model #7) using Phoenix WinNonlin 6.3 software. These data were used to simulate pharmacokinetic parameters for mice injected with doxorubicin at 0.75 mg/kg for 8 times, with the assumption that exposure in mice would be linear in this dose range. Published pharmacokinetic parameters (fit to a 3-compartment model) of human patients injected intravenously (IV) with doxorubicin at a bolus dose of 75 mg/m^2^ is presented for comparison.

**Fig 1:**
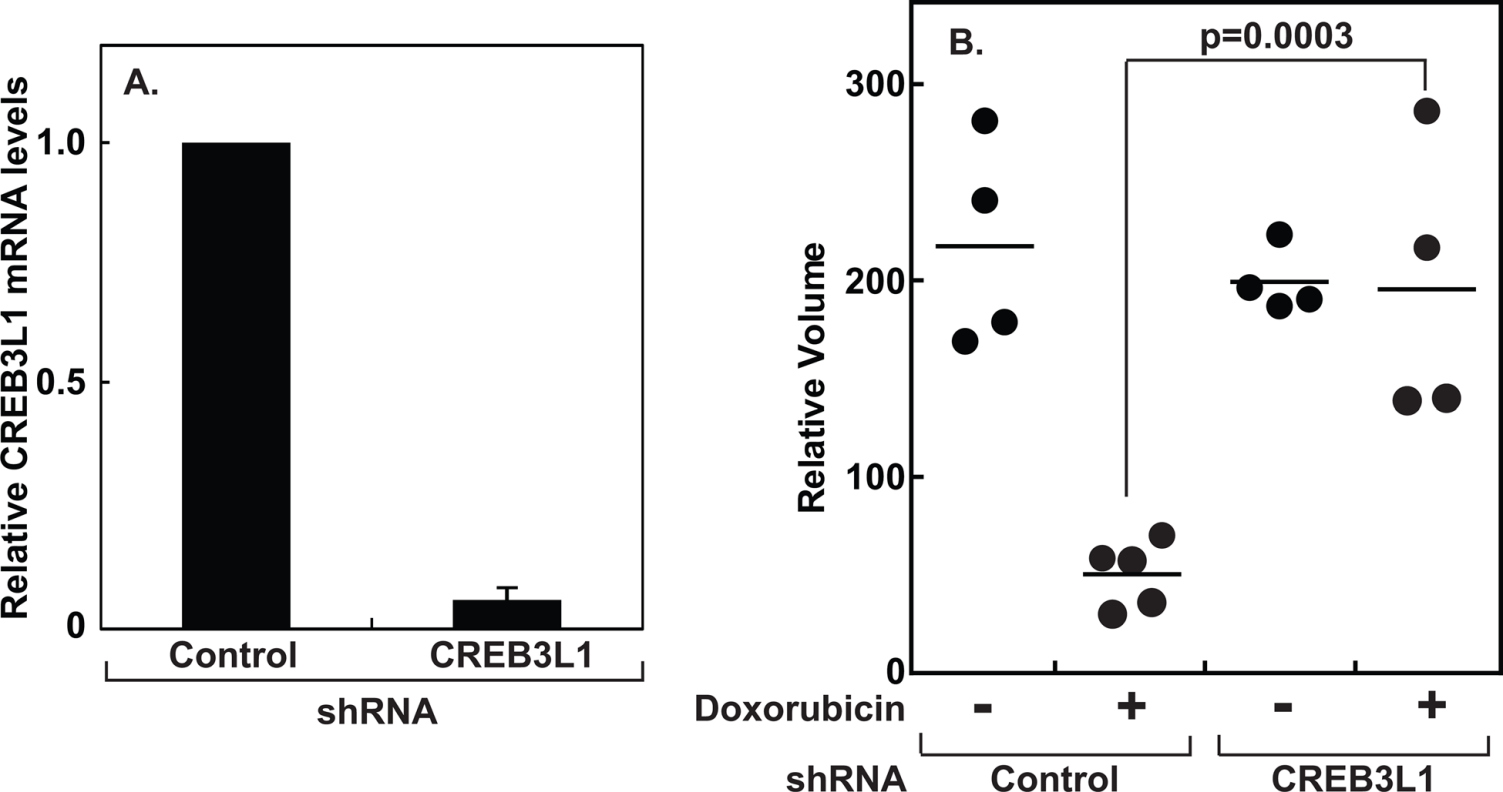
Response of xenograft tumors formed by Huh7-derived cells to doxorubicin in mice. (A) CREB3L1 mRNA in Huh7 cells stably transfected with the indicated shRNA was quantified through RT-QPCR with the value in cells transfected with the control shRNA set to 1. Results are reported as mean ± S.E. from three independent experiments. (B) Volume of the xenograft tumors formed by Huh7 cells transfected with the indicated shRNA was measured in mice 3 days after the injection of doxorubicin at 0.75 mg/kg, with the value immediately before the injection set at 100%. Unpaired two-tailed student’s t-test was performed to determine the statistical significance. Similar results were observed in two other independent experiments.

We then used this dose to determine the effect of doxorubicin on growth of human xenograft tumors. We previously reported that doxorubicin inhibited proliferation of Huh7 cells (a line of human hepatoma cells) transfected with a control shRNA but not those stably transfected with a shRNA targeting CREB3L1 when these cells were cultured *in vitro* [5]. To determine the effect of doxorubicin on growth of the cells *in vivo*, we injected these cells subcutaneously into the left flank of the back of immunodeficient mice and observed their growth following a single injection of doxorubicin. The shRNA targeting CREB3L1 reduced the expression of the mRNA by more than 90% (Fig 1A). Xenograft tumors composed of the cells transfected with either the control shRNA or that targeting CREB3L1 grew at a similar rate in the absence of doxorubicin (Fig 1B). Doxorubicin shrank the tumors formed by the cells transfected with the control shRNA but not those composed of the cells transfected with the shRNA targeting CREB3L1 (Fig 1B).

**Fig 2:**
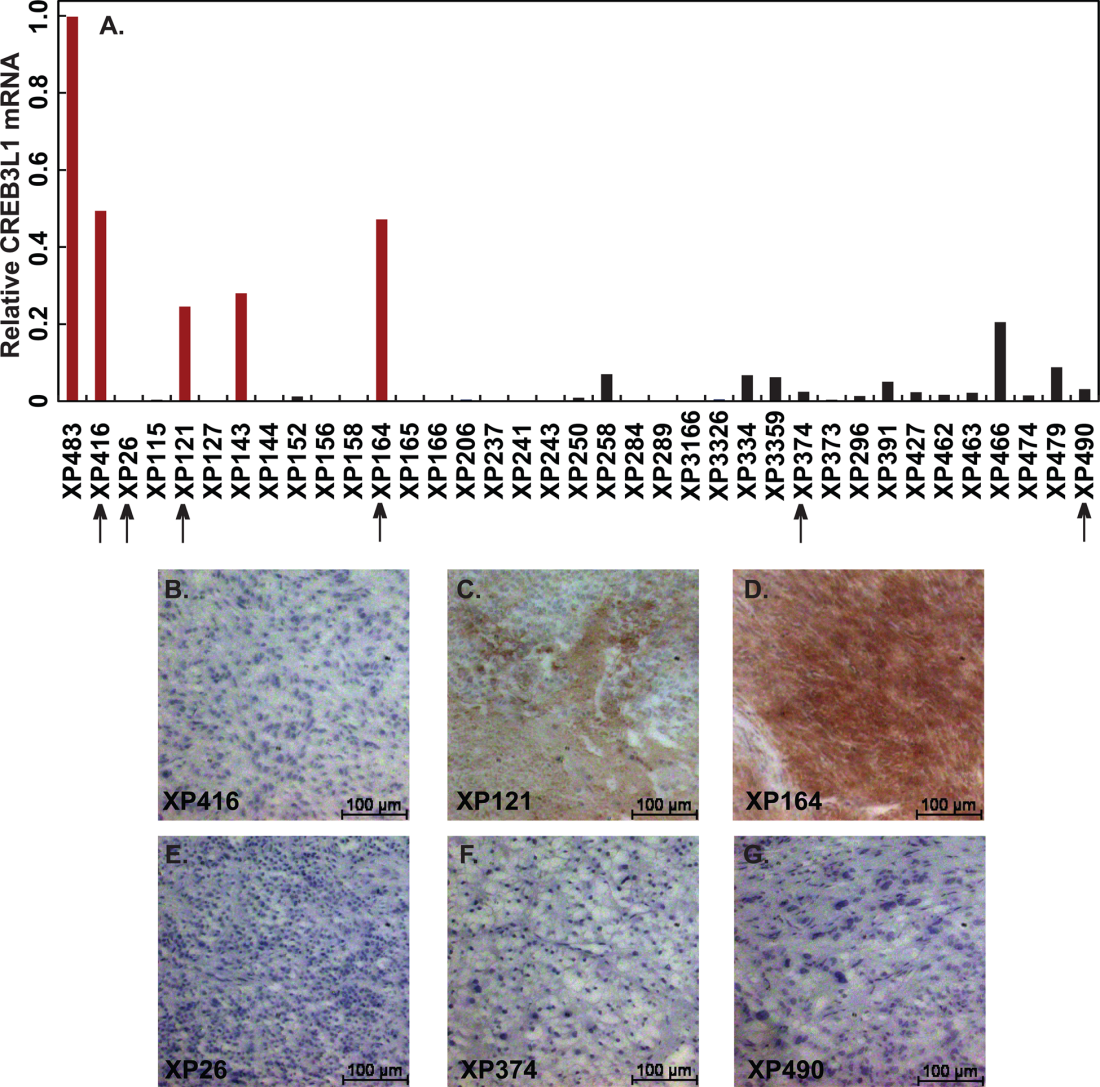
CREB3L1 expression in RCC xenograft tumors. (A) CREB3L1 mRNA in RCC tumors was quantified through RT-QPCR with the value of the highest expression set to 1. Tumors considered to have high levels of CREB3L1 expression are highlighted in red. Tumors used in xenograft experiments are indicated by arrows. (B-G) CREB3L1 protein levels in indicated tumors were measured through IHC. Scale bar: 100 μm.

We also determined the effect of doxorubicin on growth of xenograft tumors that had not been subjected to cell culture *in vitro*. For this purpose, we used a mouse xenograft model of RCC that has been shown to exhibit the same drug sensitivity as that displayed in patients [6]. We screened for *CREB3L1* expression in 37 lines of human RCC xenograft tumors passaged by injection into kidneys of immunodeficient mice [6], and identified 5 of the tumors expressed high levels of CREB3L1 mRNA (Fig 2A, red bars). In order to monitor tumor growth continuously, these tumors were transplanted subcutaneously. Among the five tumors that expressed high levels of CREB3L1 mRNA, only three (XP416, XP121 and XP164) grew efficiently after subcutaneous implantation. We thus chose these three tumors and three tumors with low or no expression of CREB3L1 (XP26, XP374, XP490) for further analysis. Since some tumor cells were previously found to express high levels of CREB3L1 mRNA but very low levels of the protein [8], we determined the amount of CREB3L1 protein in these xenograft tumors by immunohistochemistry (IHC). This analysis revealed that the amount of CREB3L1 protein was very low in XP416 (Fig 2B) even though the tumor expressed high levels of CREB3L1 mRNA (Fig 2A). The amount of CREB3L1 protein in other tumors paralleled the amount of CREB3L1 mRNA (Fig 2C–2G). Thus, high levels of CREB3L1 protein were detected in two out of the six tumors we analyzed.

**Fig 3:**
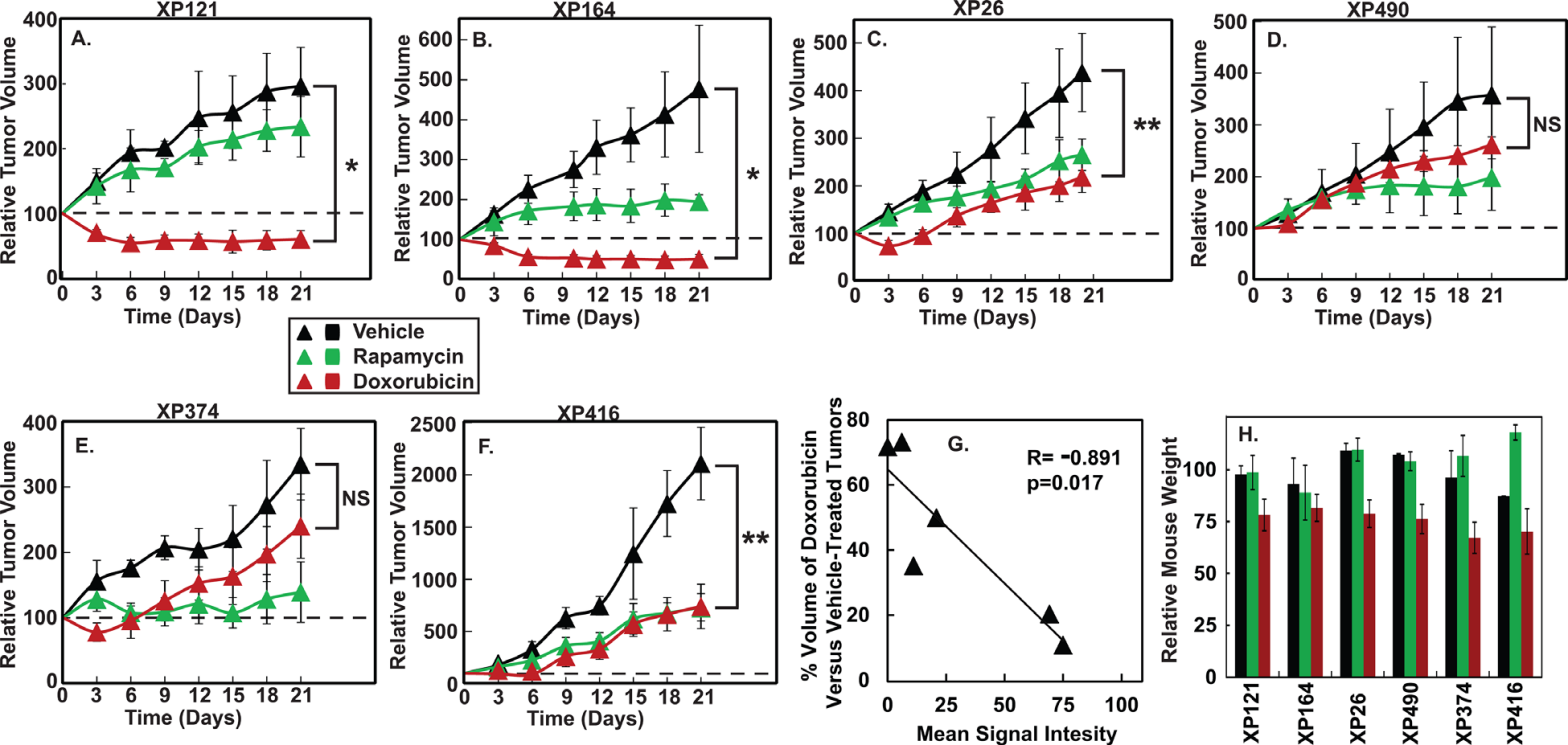
Responses of RCC xenograft tumors to doxorubicin in mice. (A-F) Volume of the xenograft tumors was measured in mice injected with doxorubicin (0.75 mg/kg), rapamycin (0.5 mg/kg) or the vehicle control once every two days. Duration of each treatment is described in detail in the Method section. The tumor volume immediately before the initial injection is set at 100%. (G) At the end of the experiments 21 days after the initial injection, the effect of doxorubicin on tumor growth expressed as the volume of doxorubicin-treated tumors as a percentage of that treated with the vehicle control was plotted against the relative intensity of CREB3L1 IHC staining in each line of the tumors (see Methods section for quantification of IHC images). Statistics was performed by Pearson analysis. (H) Weight of the mice transplanted with the indicated tumors and subjected to the indicated treatment was measured at the end of the experiments, with the value immediately before the treatment set at 100%. (A-F, H) Results are reported as mean ± S.E. from n ≥ 4 mice injected with vehicle control or doxorubicin, and n ≥ 3 mice injected with rapamycin. Unpaired two-tailed student’s t-test was performed to determine the statistical significance. *p < 10^−4^; **p < 10^−3^; NS, the difference is not statistically significant. Similar results were obtained for XP121, XP164 and XP416 in one other independent experiment.

To test the effect of doxorubicin in these tumors, we injected mice bearing the tumors with doxorubicin (0.75 mg/kg) once every two days until the mice lost 15% of their original body weight (maximal 8 injections). We monitored the tumor growth for 21 days. We also injected mice once every two days with the vehicle control or rapamycin, a standard treatment for renal cancers [9]. The two tumors with high levels of CREB3L1 expression responded much better to doxorubicin than to vehicle or rapamycin treatment. They shrank after doxorubicin treatment and did not grow back 9 days after administration of the drug was stopped (Fig 3A and 3B). In contrast, doxorubicin was less effective in inhibiting growth of the tumors that produced low levels of CREB3L1. These tumors continued to grow after doxorubicin treatment (Fig 3C–3F). When the effect of doxorubicin expressed as the volume of doxorubicin-treated tumors as a percentage of that of the vehicle-treated control was plotted against the relative levels of CREB3L1 expression in tumors obtained through quantification of the IHC results shown in Fig 2B–2G, a clear correlation between higher levels of CREB3L1 protein in RCCs and increased doxorubicin sensitivity was observed (p = 0.017 by Pearson analysis) (Fig 3G). Upon doxorubicin treatment, mice transplanted with the tumors that expressed high levels of CREB3L1 did not lose more weight than those transplanted with tumors producing low levels of the protein (Fig 3H). These results suggest that the effect of doxorubicin on tumors that produced high levels of CREB3L1 was not caused by nonspecific toxicity associated with chemotherapy.

**Fig 4:**
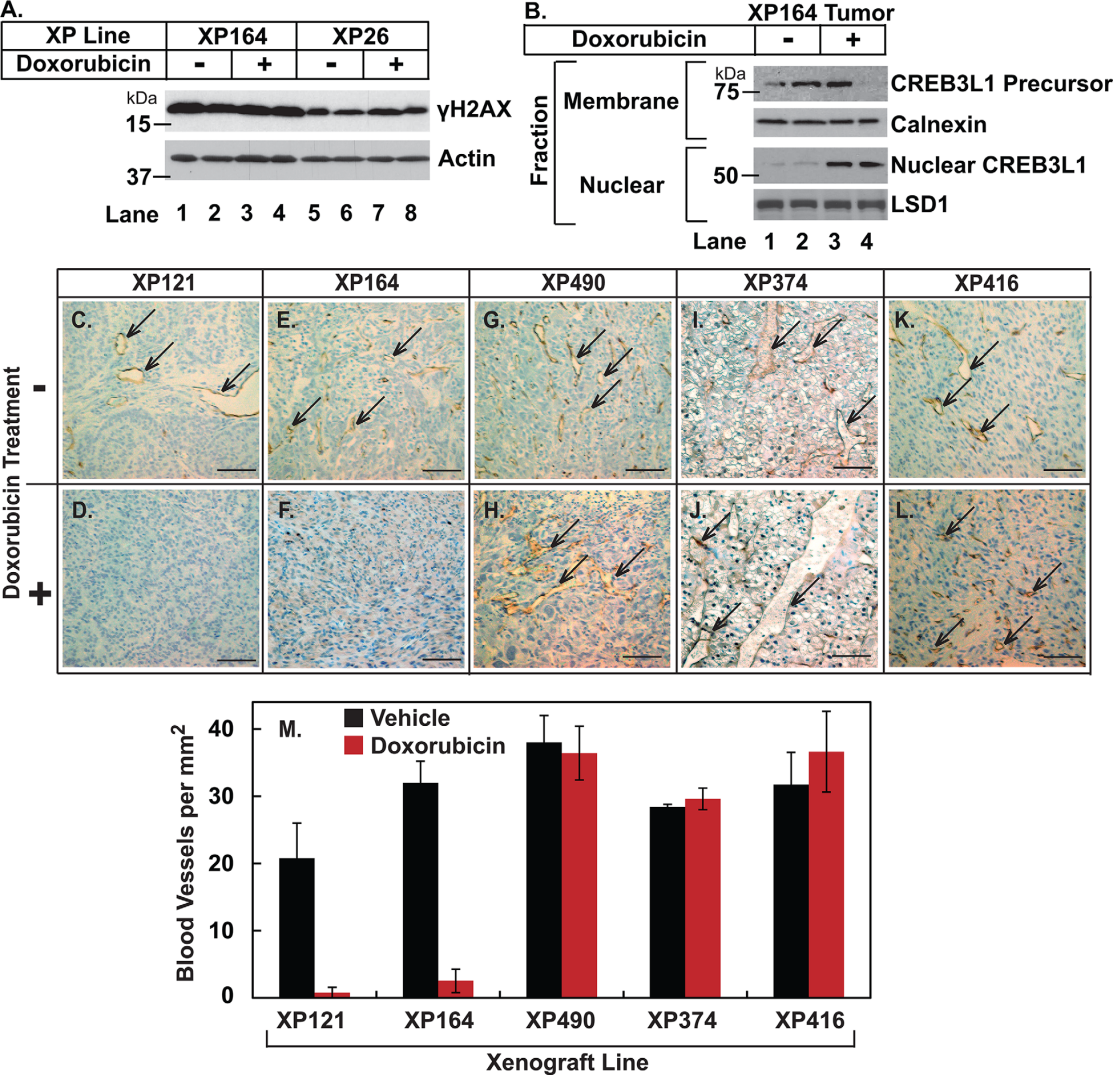
Doxorubicin inhibits growth of tumors expressing high levels of CREB3L1 by stimulating cleavage of CREB3L1. (A and B) Whole cell lysate (A) or indicated fractions (B) of the RCC xenograft tumors harvested 3 days after the initial injection (2 mice for each tumor) were subjected to immunoblot analysis with the indicated antibodies. Calnexin, lysine-specific demethylase 1 (LSD1) and actin served as loading controls. (C-L) Tumors harvested 21 days after the initial injection were subjected to IHC with anti-endomucin. Representative blood vessels marked by endomucin staining are indicated by arrows. Scale bar: 100 μm. (M) Quantification of blood vessel density in each line of the tumors determined by IHC of endomucin. For each individual tumor, five independent images were taken for calculation. For each line of tumors, results are reported as mean ± S.E. from n ≥ 4 tumors taken from different mice.

To determine whether doxorubicin inhibited growth of the tumors that expressed high levels of CREB3L1 by inducing DNA damage, we harvested the xenograft RCC tumors on day 3 after two injections of doxorubicin to measure the amount of histone γH2X, a marker for DNA breaks, by immunoblot analysis. Histone γH2X was present in tumors from mice injected with the vehicle control regardless of CREB3L1 expression (Fig 4A, lanes 1, 2, 5 and 6), and injection of doxorubicin did not enhance the amount of γH2X (Fig 4A, lanes 3, 4, 7 and 8). We also performed immunoblot analysis to detect cleavage of CREB3L1 in xenograft tumors that expressed high levels of the protein. The cleaved nuclear form of CREB3L1 was detectable in tumors transplanted in mice injected with doxorubicin (Fig 4B, lanes 3 and 4) but not in those injected with the vehicle control (Fig 4B, lanes 1 and 2).

The nuclear CREB3L1 was reported to inhibit tumor growth by activating transcription of genes that inhibit angiogenesis [10]. We thus performed IHC analysis to detect blood vessels by staining the xenograft tumors with an antibody against endomucin, a marker for endothelial cells. In tumors that produced high levels of CREB3L1, blood vessels were visible in tumors from mice injected with the vehicle control but were barely detectable in tumors from those injected with doxorubicin (Fig 4C–4F). In contrast, blood vessels were readily detectable in tumors that expressed low levels of CREB3L1 regardless of the doxorubicin treatment (Fig 4G–4L). Quantification of the IHC results revealed that doxorubicin reduced the density of blood vessels in tumors that expressed high levels of CREB3L1 by more than 90% (Fig 4M). In contrast, the density of blood vessels in tumors that expressed low levels of CREB3L1 was not affected by doxorubicin treatment (Fig 4M).

**Fig 5:**
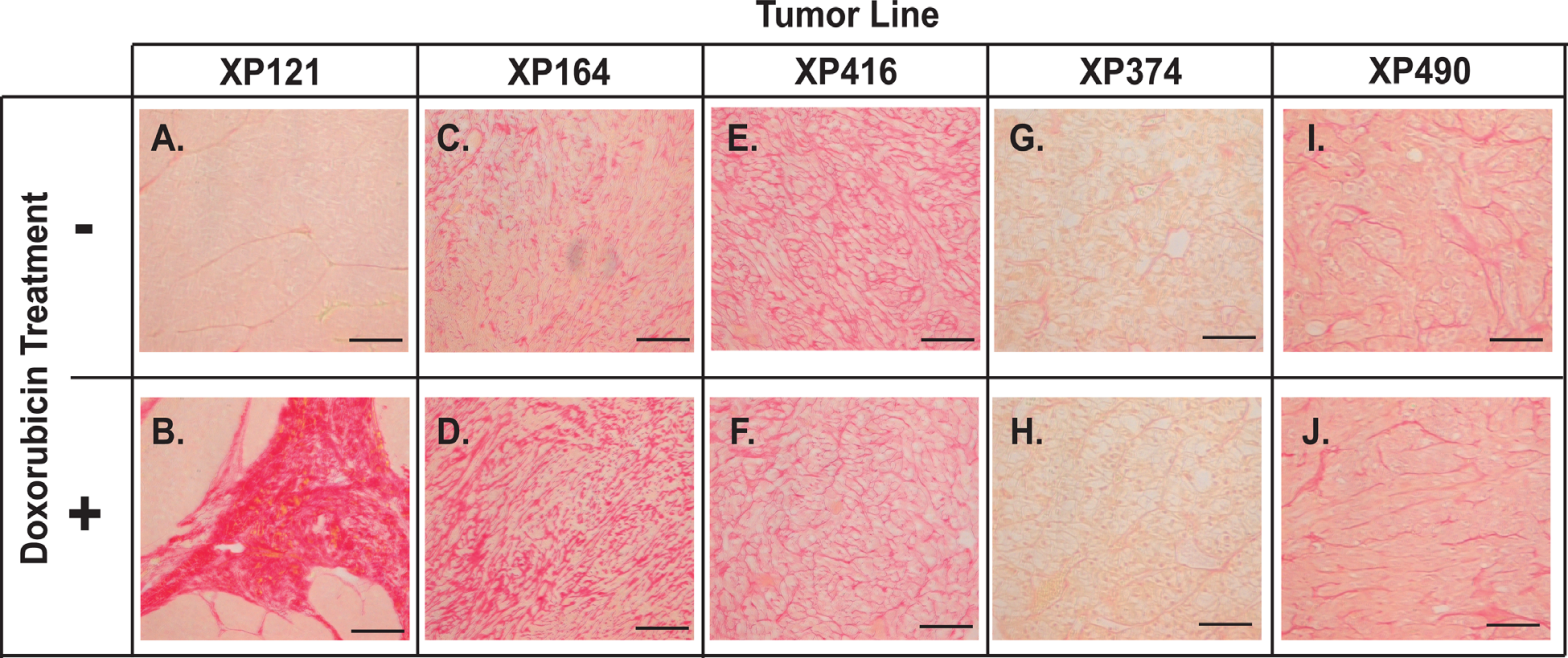
Doxorubicin induces collagen deposition in tumors expressing high levels of CREB3L1. Pico Sirius Red staining of collagen-containing matrix in RCC xenograft tumors harvested 21 days after the initial injection of doxorubicin as indicated. Scale bar: 100 μm.

In addition to genes that inhibit tumor growth, the nuclear from of CREB3L1 also activates genes required for assembly of collagen-containing extracellular matrix [5,11]. We thus performed histology analysis of the xenograft tumors with Picro-Sirius Red to detect collagen fibers deposited in the tumors. The intensity of collagen staining was enhanced by doxorubicin in tumors that expressed high levels of CREB3L1 (Fig 5A–5D) but not in those that expressed low levels of the protein (Fig 5E–5J). These results suggest that doxorubicin activates the transcription programs induced by nuclear CREB3L1 *in vivo*.

**Fig 6:**
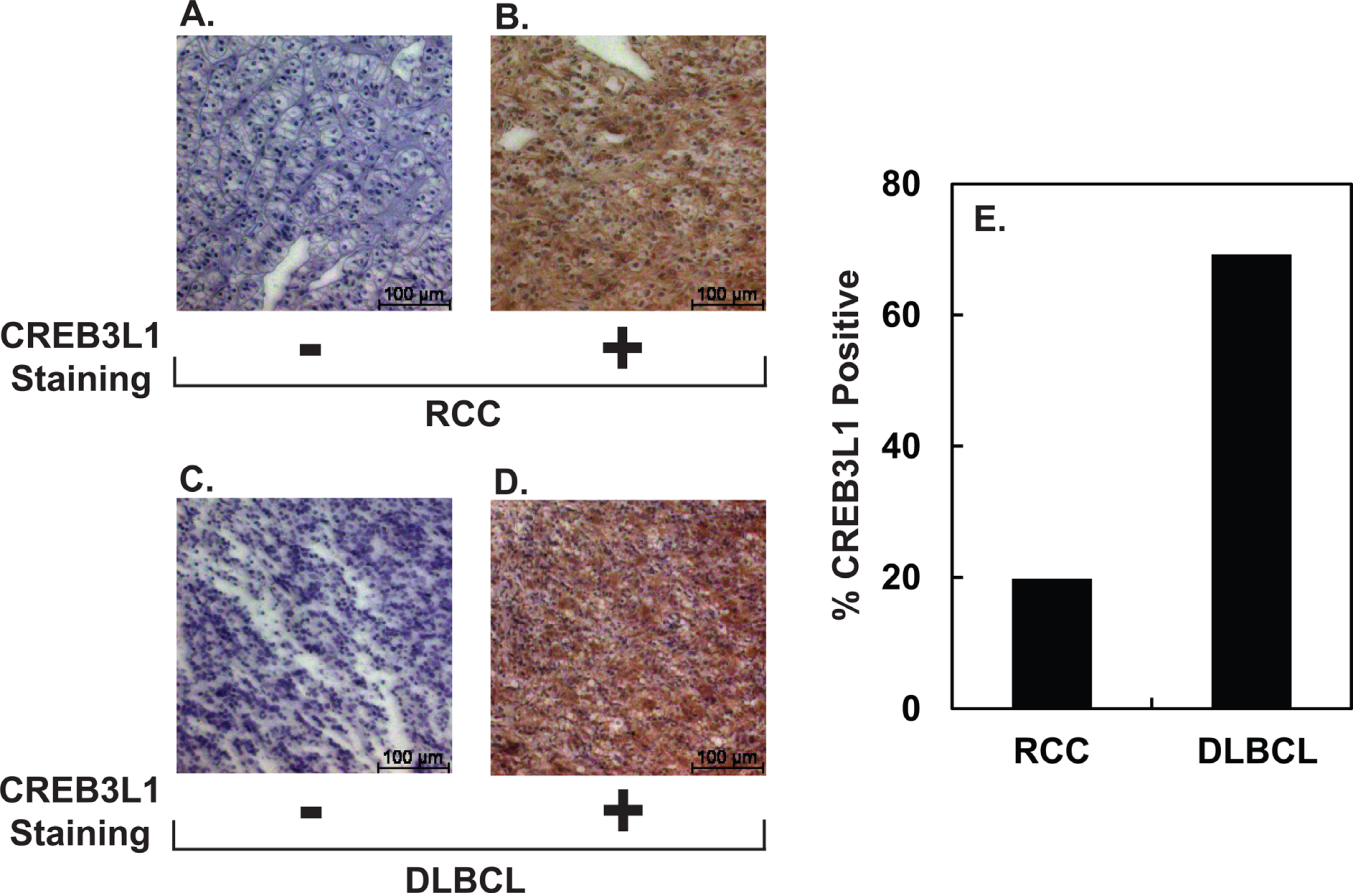
CREB3L1 expression in RCC and DLBCL. (A-D) Representative IHC images of sections of the indicated tumors stained negatively or positively with anti-CREB3L1. Scale bar: 100 μm. (E) Percentage of indicated tumors stained positively with anti-CREB3L1.

The results in Fig 3A show that the percentage of RCC tumors that express CREB3L1 is quite low. This observation may explain why doxorubicin is not approved to treat RCC. On the other hand, doxorubicin is used as the primary chemotherapeutic reagent to treat DLBCL [12]. According to our hypothesis, a substantial fraction of DLBCL should express CREB3L1. To test this hypothesis, we used IHC to measure CREB3L1 expression in biopsies from 29 RCC and 14 DLBLC patients. Examples of the positive and negative staining results for each tumor were shown in Fig 6A–6D. Among the primary RCC, 19% were stained positively with anti-CREB3L1 (Fig 6E). In contrast, 70% of DLBLC showed positive staining (Fig 6E).

To expand these data we analyzed CREB3L1 expression using Oncomine, an online tool to compare levels of mRNA expression in thousands of primary tumors deposited in the database [13]. This analysis revealed that osteosarcoma, malignant fibrous histiocytoma and ovarian mucinous adenocarcinoma have the highest levels of CREB3L1 mRNA (Table 2). Doxorubicin is used as the standard treatment for two of these tumors (osteosarcoma and malignant fibrous histiocytoma) [14,15]. Ovarian mucinous adenocarcinoma accounts for less than 15% of ovarian cancers [15]. Doxorubicin is used as a non-standard reagent to treat ovarian cancer, but its effect specifically on ovarian mucinous adenocarcinoma has not been determined [16]. At the other end of the spectrum, we identified chondrosarcoma, RCC, squamous cell lung cancer, brain cancers and small cell lung carcinoma as the cancers with the lowest levels of CREB3L1 mRNA (Table 2). Doxorubicin is not clinically approved to treat these cancers except for small cell lung carcinoma for which doxorubicin is sometimes included as a non-standard treatment reagent [17] (Table 2).

**Table 2:** Clinical application of doxorubicin to cancers with the highest or lowest levels of CREB3L1 expression.

	Cancer Type	p-Value	Fold	Clinical Application of Doxorubicin
Highest	Osteosarcoma	2.2E-08	3.3	Standard Treatment
CREB3L1	Malignant Fibrous Histiocytoma	3.6E-06	20.6	Standard Treatment
Expression	Ovarian Mucinous Adenocarcinoma	6.4E-06	4.5	Non-Standard Treatment^A^
	Chondrosarcoma	5.0E-05	-7.0	Not Used
Lowest	RCC	8.0E-74	-4.2	Not Used
CREB3L1	Squamous Cell Lung Cancer	2.7E-06	-3.0	Not Used
Expression	Braun Cancers	2.3E-13	-7.3	Not Used
	Small Cell Lung Carcinoma	1.8E-10	-21.4	Non-Standard Treatment

Cancers with the highest or lowest levels of CREB3L1 mRNA were identified through bioinformatics analysis of the Oncomine database. To search for these cancers, p-values were set to less than 10^−4^ to minimize variation in CREB3L1 expression among individual tumors in a given type of cancer, and fold of changes were set to more than 3 to select tumors in which levels of CREB3L1 mRNA is at least 3 times higher or lower than the average value of the mRNA across all the tumors deposited into the database. Tumors were ordered by Oncomine according to combined evaluation of fold of changes and p-values. American Cancer Society (www.cancer.org) and National Cancer Institute (www.cancer.gov) were consulted to obtain information regarding clinical application of doxorubicin to each cancer.

^A^ Doxorubicin is used as a non-standard treatment for ovarian cancers in general, but its effect on ovarian mucinous adenocarcinoma has not been determined.

## Discussion

The current study demonstrates that production of CREB3L1 in tumor cells is crucial to determine their sensitivity to doxorubicin. Using mouse xenograft models, we showed that doxorubicin shrank tumors expressing high levels of CREB3L1 but not those that had low or no expression of the protein. This conclusion was further supported by our observation that the percentage of the tumors expressing CREB3L1 is higher in cancer types empirically determined to be responsive to doxorubicin treatment (e.g. DLBLC) as opposed to those resistant to doxorubicin (e.g. RCC). When we analyzed DLBLC, we also tried to determine whether higher expression of CREB3L1 in tumor biopsies before chemotherapy was correlated to better responses to later treatment with doxorubicin. The result was inconclusive because these cancer patients were not treated with doxorubicin alone but with chemotherapy regime containing multiple drugs.

The results from the current study suggest that CREB3L1 may serve as a biomarker for doxorubicin sensitivity. By measuring the levels of CREB3L1 in tumor biopsies before chemotherapy and using doxorubicin only in patients with cancers expressing CREB3L1, the response rate of doxorubicin may be markedly improved for cancers such as breast cancer that are treated with chemotherapeutic regime containing doxorubicin but with a low response rate [18]. This approach may also enable doxorubicin to treat rare subsets of cancers like RCC that are not currently treated with the drug. The 10H1 monoclonal antibody against CREB3L1 developed in the current study makes it possible to measure CREB3L1 expression in tumor biopsies. We have demonstrated in the current study that this antibody can be used to measure CREB3L1 expression in tumor cells from biopsies of RCC and DLBLC.

An important finding in the current study is that doxorubicin shrank xenograft tumors expressing high levels of CREB3L1 at a dose much lower than that currently applied to patients without inducing additional DNA damage. Thus, at least for tumors expressing high levels of CREB3L1, doxorubicin appears to inhibit tumor growth by triggering proteolytic activation of CREB3L1 rather than stimulating DNA damage. It was reported previously that doxorubicin at a dose much higher than that was used in the current study caused cardiac toxicity in mice as a result of DNA damage [19]. It will be interesting to determine whether tumors expressing high levels of CREB3L1 can be effectively treated with lower doses of doxorubicin to alleviate the cardiac toxicity associated with the chemotherapy.

## Methods

### Materials

We obtained mouse anti-γH2AX from Millipore; peroxidase-conjugated secondary antibodies from Jackson ImmunoResearch; rabbit anti-Actin and Doxorubicin (Cat# D1515-10MG) from Sigma-Aldrich; Rapamycin from LC Laboratories; rabbit anti-LSD1 from Cell Signaling; mouse anti-calnexin from Enzo Life Sciences. A mouse monoclonal antibody against human CREB3L1 (10H1) was generated by immunizing mice with synthesized polypeptides corresponding to amino acids 7–41 of human CREB3L1.

### Cell Culture

Huh7 cells stably transfected with a control shRNA or that targeting CREB3L1 were generated as previously described [5]. These cells were maintained in Dulbecco's modified Eagle's medium (high glucose) supplemented with 10% fetal calf serum, 100 U/ml penicillin, 100 μg/ml streptomycin sulfate and 10 μg/ml puromycin. All cells were incubated in monolayers at 37°C in 5% CO_2_.

### Pharmacokinetic Analysis of Doxorubicin

Adult female nu/nu mice were injected intraperitoneally with doxorubicin at 5 mg/kg in a total volume of 0.2 ml. Mice were sacrificed at varying times (5 min—72 h) following the injection for collection of plasma. The amount of doxorubicin in the plasma was measured by the Preclinical Pharmacology Core Lab in UTSW. A standard noncompartmental analysis with sparse sampling was performed for initial data evaluation using plasma doxorubicin concentration-time profiles. The maximum plasma drug concentration and time to reach the maximal concentration were obtained directly from the experimental data. Half-life, area under the concentration time curve from time zero to the last measured time point (AUC, calculated by linear trapezoidal method) were calculated using the noncompartmental analysis tool in Phoenix WinNonlin 6.3 (Pharsight, Mountain View, CA). The data were also fit to various WinNonlin compartmental pharmacokinetic models before settling on model #7 (Two-compartment with bolus input and first-order output; micro-constants as primary parameters) to predict doxorubicin levels in mice receiving injection of the drug at 0.75 mg/kg once every 2 days for a total of 8 injections. Goodness-of-fit was assessed by Akaike Information Criterion and visual inspection of residuals and fitted curves.

### Xenograft Tumor Analysis

For experiments shown in Fig 1, Huh7 cells stably transfected with a control shRNA or that targeting CREB3L1 were seeded at 1 × 10^6^ cells per 150 mm dish. Cells were harvested 4 days later and resuspended in PBS at 1 x 10^9^ cells/ml. Adult female nu/nu mice were injected subcutaneously with 1 x 10^8^ cells. Once the tumors reached 100 mm^3^, the mice were injected intraperitoneally (IP) with a single dose of doxorubicin at 0.75 mg/kg or the vehicle control at the same volume. The tumor volume was measured 3 days after the injection and compared to that measured immediately before the injection.

For experiments shown in Fig 3, NOD/SCID mice were transplanted subcutaneously with human RCC tumors passaged in mice as previously described [20]. Once the tumors reached at least 50 mm^3^, mice were subject to IP injection once every two days with vehicle (PBS), doxorubicin (0.75 mg/kg) or rapamycin (0.5 mg/kg). Injections of doxorubicin were ceased when mice lost 15% of their original body weight (8 injection maximally). Vehicle injections followed the same schedule of doxorubicin injections. Rapamycin was injected until the end of the experiments 21 days after the initial injection. Tumor volume and mice weight was measured once every three days for the duration of the experiments. Tumors were harvested for immunoblot and histology analysis.

### IHC and Histology Analysis

Tumors fixed in 4% formalin were submitted to the Molecular Pathology Core at UTSW for paraffin sectioning and subsequent Pico Sirius Red and endomucin staining. Tumor and benign tissues in tumor biopsies were determined by Weina Chen, a board-certified pathologist, through H&E staining. Parallel slides were subjected to IHC of CREB3L1 to determine CREB3L1 expression in the tumors. For this purpose, paraffin-embedded sections were treated with xylenes, washed with ethanol and water, and blocked with 3% hydrogen peroxide for 5 min. After washing with PBS, antigen retrieval was performed using the Retriever 2100 Buffer U (Electron Microscopy Sciences) according to the manufactures’ directions. Sections were then blocked with Blocking Buffer (1% Horse Serum in PBS) for 1 hr, incubated with 17 μg/ml 10H1 overnight in Blocking Buffer at 4°C, washed three times with PBS, and developed with the DAB staining procedure using the Vectastain Elite ABC Kit and DAB Peroxidase (HRP) Substrate Kit (Vector Laboratories). Counterstaining was performed with hematoxylin. Bright field images for all histology analyses were taken using a Zeiss Observer Z1 microscope using AxioVision software. Any samples showing signal greater than that of the controls stained in the absence of the primary antibody (10H1) are considered to be positive for CREB3L1 expression.

### Immunoblot Analysis

For analysis of CREB3L1 cleavage, cells were fractionated into nucleus and membrane fractions as previously described [21]. Immunoblot analysis was carried out as previously described [5] except that CREB3L1 was detected by 10H1 (1:1000 dilution).

### RT-QPCR

RT-QPCR was performed as previously described [22]. The relative amounts of RNAs were calculated through the comparative cycle threshold method by using human 36B4 mRNA as the invariant control.

### Bioinformatics Analysis

The Oncomine database was searched on 12/29/2014 to compare mRNA expression using a cancer versus cancer analysis with the following cutoffs: p-values were set to less than 10^−4^ to minimize variation in CREB3L1 expression among individual tumors in a given type of cancer; and fold of changes were set to more than 3 to select tumors in which levels of CREB3L1 mRNA is at least 3 times higher or lower than the average value of the mRNA across all the tumors deposited into the database. Tumors were ordered by Oncomine according to combined evaluation of fold of changes and p-values. Cell lines cultured *in vitro* were excluded from the analysis. The sample size for osteosarcoma, malignant fibrous histiocytoma, ovarian mucinous adenocarcinoma, chondrosarcoma, RCC, squamous cell lung cancer, brain cancers and small cell lung carcinoma is 96, 51, 241, 36, 1911, 73, 288 and 203, respectively.

### Statistics

For result shown in Fig 3G, the signal intensity of CREB3L1 IHC staining without hematoxylin counterstaining was quantified by ImageJ software. Pearson analysis was performed to determine the correlation between the effects of doxorubicin on tumor growth versus levels of CREB3L1 expression in tumor cells measured through IHC.

### Ethic Statement

All human samples were collected in UT Southwestern Medical Center (UTSW) according to protocols approved by UTSW institutional review board (STU 022011-057). Written informed consent was received from participants prior to inclusion in the study. All animal experiments were approved by UTSW institutional animal care and use committee (2011-0192). The mice were sacrificed by inhalant anesthesia overdose (isoflurane).
